# Attitude of patients, healthcare professionals, and noninjured lay persons towards online video instructions on mild traumatic brain injury: a cross-sectional study

**DOI:** 10.1186/s12245-017-0151-x

**Published:** 2017-07-27

**Authors:** Amber E. Hoek, Maaike van den Hamer, Carianne K. Deelstra, Ed F. van Beeck, Diederik W. J. Dippel, Juanita A. Haagsma, Pleunie P. M. Rood

**Affiliations:** 1 Department of Emergency Medicine, Erasmus University Medical Center, P.O. Box 2040, 3000 CA Rotterdam, The Netherlands; 2 Department of Public Health, Erasmus University Medical Center, P.O. Box 2040, 3000 CA Rotterdam, The Netherlands; 3 Department of Neurology, Erasmus University Medical Center, P.O. Box 2040, 3000 CA Rotterdam, The Netherlands

**Keywords:** Mild traumatic brain injury, Emergency department, Discharge instructions, Video

## Abstract

**Background:**

The objective of this study was to determine the attitude of patients, healthcare professionals, and noninjured lay persons towards adding a video with discharge instructions to patient care for patients with mild traumatic brain injury (MTBI). A survey was conducted at the emergency department (ED). Participants consisted of MTBI patients (*n* = 50), healthcare professionals (*n* = 50), and noninjured lay persons (*n* = 50). The participants viewed a video with discharge instructions on MTBI and filled out a questionnaire that measured their attitude towards the use of a video as part of discharge instructions.

**Findings:**

Nearly all healthcare professionals (94%) and 70% of the noninjured lay persons considered the video to be a valuable addition to oral discharge instructions. For 84% of patients, verbal information from the doctor is of importance. And, 50% of patients would like to receive additional video discharge instructions.

**Conclusions:**

The majority of noninjured lay persons and healthcare professionals and half of the MTBI patients consider a video with discharge instructions to be a valuable addition to patient care. Video discharge instructions are a relative low-cost measure that could enhance patient care at the ED, provided that this does not compromise the personal contact between patient and healthcare professional.

## Introduction

Mild traumatic brain injury (MTBI), or concussion, often leads to persistent symptoms. These symptoms, such as headaches, mild cognitive problems, and dizziness, may last for weeks or even months after the concussion [1–3].

Evidence suggests that providing patients with discharge instructions containing adequate educational information on MTBI can help reduce or even prevent post-concussion symptoms, particularly early after the injury [4]. Patients remember discharge instructions better when they receive written instructions additional to oral information only [5]. However, a prerequisite for the effectiveness of written discharge instruction is a sufficient literacy level of the recipient. For some treatments in the emergency department (ED) video discharge instructions have shown to improve comprehension and recall of key points [6, 7]. However, for patients with brain concussion, potentially suffering from headache and cognitive problems, this is unknown.

An important step in finding the most effective way of providing patient information on MTBI is to assess the feasibility and acceptability of an online video with discharge instructions.

The objective of this study was to determine the attitude of patients, healthcare professionals, and noninjured lay persons towards adding a video with discharge instructions to patient care for patients with MTBI.

## Methods

A survey study was conducted at the ED of a tertiary center in Rotterdam, the Netherlands, between November 2014 and July 2015. The aim was to include 50 MTBI patients, 50 healthcare professionals, and 50 noninjured lay persons. The study subjects consisted of a convenience sample of MTBI patients, healthcare professionals, and noninjured lay persons. The noninjured lay persons were recruited from a social network by contacting them personally or by email. Snowball sampling was used for the recruitment strategy, i.e., participants were asked to recruit future participants among their contacts. The selection criterion was that the noninjured lay persons were not engaged in any medical profession. The healthcare professionals consisted of nurses and doctors. Selection criteria for the healthcare professionals were that they had to be entrusted with the care for MTBI patients and were not involved in the study. Healthcare professionals were recruited during their shift at the ED or the neurology department. The selection criteria for MTBI patients were treatment at the ED, aged 18 years and older, a Glasgow Coma Scale (GCS; a neurological scale ranging from 3 to 15 that is based on eye, motor, and verbal responses of the patient) greater than 13 at first contact, and post-traumatic loss of consciousness of less than 30 min (indicated in the history of the patients). MTBI patients were excluded if they did not master the Dutch language, had an intracranial abnormality on the CT-scan, a focal neurological deficit, or if they were unable to give informed consent. Patients were recruited by one of the researchers according to a rotating schedule representing all shifts and days of the week.

Once participants decided to participate in the study, they were shown a video with discharge instructions for MTBI (https://drive.google.com/file/d/0B90l2el_JbUtVlhWdk1TM3ltdkE/edit?usp=sharing). The information in this video is based on the national guideline for management of mild head injury which includes advisory information for patients. The discharge instructions shown in the video provided background information on MTBI, information about common symptoms, typical course of recovery, advice about how to manage or cope with symptoms, gradual reintegration to regular activities, and when to contact a medical expert. Healthcare professionals and noninjured lay persons either watched the video on a research computer (individually or in small groups) or at home through the online link. Hereafter the healthcare professionals and noninjured lay persons filled out the questionnaire individually, on paper, or digitally. Patients watched the video at the ED after their treatment was finished, before discharge from the ED when they had a GCS of 15. They filled out the questionnaire immediately after watching, at the ED. The questionnaire administered to the MTBI patients and noninjured lay persons which was developed in agreement with a psychologist and an emergency physician, consisted of questions on age, sex, and educational level (high education level: finished at least higher secondary education) and nine questions concerning the video and their attitude towards the applicability and value of the use of adding the video with discharge instructions to patient care. The questionnaire administered to the healthcare professionals consisted of questions on age, sex, position, and 11 questions on the video and their attitude towards the applicability and value of the use of adding the video with discharge instructions to patient care. The questionnaire also included open ended questions which asked the participants to explain their answer. Ethical approval was obtained from the research ethics committee of the study center before the initiation of the study (MEC-2015-175). All MTBI patients gave written informed consent before inclusion.

Data was analyzed with descriptive statistics (frequencies and crosstabs with chi-square testing) in statistical package for social sciences (SPSS) 21.0.

## Findings

### MTBI patients

**Fig. 1:**
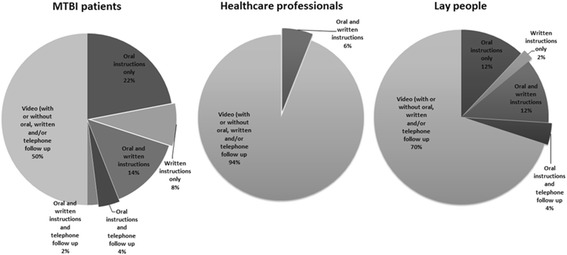
Preferred mode of discharge instructions

During the study period 78 MTBI patients were seen at the ED, of which 58 met the inclusion criteria. Of these patients, 50 (86.2%) were willing to participate in the study. The median age of the MTBI patients was 46.5 years [range: 18–93] (Table 1). MTBI patients were mainly elderly women (age > 50 years) or young men (age ≤ 50 years). Verbal information from the doctor is deemed important by 84% of patients, of whom 74% (*n* = 31) would like to receive additional written, telephone, or video discharge instructions 68.0% (*n* = 34) stated that they would watch the video at home. Half of the patients (*n* = 25, 50%) indicated that they preferred the video to be part of their discharge instructions (Fig. 1). Age, sex, and educational level did not differ between patients who did or did not prefer video discharge instructions.

**Table 1:** Patient characteristics

Participant characteristics			
	MTBI patients	Healthcare professionals	Noninjured lay persons
	(*n* = 50)	(*n* = 50)	(*n* = 50)
Male	27 (54%)	19 (38%)	26 (52%)
Age (median and range)	46.5 [18–93]	30.5 [23–61]	48 [21–87]
High level of education*	14 (28%)	–	18 (36%)

*High education level: finished at least higher secondary education

### Healthcare professionals

Fifty healthcare professionals participated in the study. The median age was 30.5 years [range 23–61] (Table 1). 94.0% (*n* = 47) considered the video to be a valuable addition to their discharge instructions and all would advise patients to watch the video (Fig. 1). 46.0% (*n* = 23) was in favor of giving patients the link to the online video so they can watch the video at home. No statistically significant difference was found in preference for video discharge instructions for gender or age of the healthcare professionals.

### Noninjured lay persons

Fifty noninjured lay persons were included with a median age of 48.0 years [range 21–87] (Table 1). Seventy percent (*n* = 35) considered the video to be a valuable part of discharge instructions (Fig. 1). No statistically significant difference was found in preference for video discharge instructions for gender or age. However, noninjured lay persons with a high educational level would like to receive video discharge instructions significantly more often than noninjured lay persons with a low educational level (*p* < 0.05).

## Discussion and conclusion

### Discussion

This study showed that the majority of healthcare professionals and noninjured lay persons consider a video with discharge instructions to be a valuable addition to patient care.

Half of the of MTBI patients believed that a video could be a valuable part of their discharge instructions. Our study showed that 84% of the patients indicated that they wanted to receive oral discharge instructions, with or without additional alternative methods. We speculate that the main reason for not wanting a video may be that they place high value on personal contact with medical professionals.

Remarkable is that compared to other studies, we found less patients would be content with the video as part of their discharge instructions [8]. An important difference with these studies was that the patients could watch the video at home. An online video would give patients (and their significant others) the opportunity to watch discharge instructions at a time when symptoms have reduced. Moreover, they can watch the video multiple times and in a less stressful environment than the ED, increasing comprehension and recall of key discharge instructions. Also, these studies did not study specifically MTBI patients. The sustained brain injury may influence the willingness and capability to watch a video.

A necessary condition for offering online video with diagnosis-specific discharge instructions is internet access among the target group. In the Netherlands, 97% of the inhabitants have internet access [9].

Important to note is that the video used in this study showed a doctor explaining MTBI and the consequences and some parts included written text, but the video did not contain animations. This may have affected our results. A recent study concluded that spoken animations may be the best way to explain complex health information to people with low health literacy [10]. Hence, the proportion of people who would like to watch a video as part of their discharge instructions may be higher if the video includes animations. More research is needed to investigate the effectiveness of video discharge instructions with animations, both in patients with MBTI and other diseases and syndromes.

### Conclusions

The majority of noninjured lay persons and healthcare professionals, and half of the MTBI patients consider a video with discharge instructions to be a valuable addition to patient care. Video discharge instructions are a relative low-cost measure that could enhance patient care at the ED, provided that this does not compromise the personal contact between patient and healthcare professional.

### Clinical implications

From a professional perspective, video discharge instructions could enhance patient care at the ED, but this should be embedded in counseling approaches tailored to different preferences of patients.

## Abbreviations

ED: Emergency department
GCS: Glasgow coma scale
MTBI: Mild traumatic brain injury
SPSS: Statistical package for social sciences
